# Variation in Ovine *KRTAP13-3* and Its Association with Wool Characteristics in Chinese Tan Sheep

**DOI:** 10.3390/ani15071069

**Published:** 2025-04-07

**Authors:** Lingrong Bai, Huitong Zhou, Jinzhong Tao, Jon G. H. Hickford

**Affiliations:** 1 International Wool Research Institute, Faculty of Animal Science and Technology, Gansu Agricultural University, Lanzhou 730070, China; lingrong.bai@lincolnuni.ac.nz (L.B.); huitong.zhou@lincoln.ac.nz (H.Z.); tao_jz@nxu.edu.cn (J.T.); 2 Gene-Marker Laboratory, Faculty of Agriculture and Life Sciences, Lincoln University, Lincoln 7647, New Zealand; 3 College of Animal Science and Technology, Ningxia University, Yinchuan 750021, China

**Keywords:** keratin-associated protein 13-3 gene, polymorphism, FDSD, CVFD, heterotypic hair, Chinese Tan sheep

## Abstract

Understanding the genetic factors that influence wool quality is essential for enhancing wool quality and uniformity. This study investigated the *KRTAP13-3* gene in Chinese Tan sheep, a breed known for its unique wool characteristics. We analysed 232 sheep and revealed five previously identified sequence variants of *KRTAP13-3* and then modelled to ascertain whether there was any association between the nucleotide sequence variation and variation in mean fibre diameter (MFD), fibre diameter standard deviation (FDSD), coefficient of variation of fibre diameter (CVFD), and mean fibre curvature (MFC). Twelve genotypes were observed, with the five variants having frequencies that ranged from 64.0% to 1.1%. Among the four variants with frequencies above 5%, nucleotide sequence variation was associated with heterotypic hair fibre diameter variation. The most common variant (*A*) was linked to increased FDSD and CVFD, while two other variants (*B* and *D*) revealed trends towards being associated with decreased CVFD. No associations were found with variation in the fine wool fibres from the Tan sheep. This suggests that *KRTAP13-3* plays a role in regulating heterotypic hair fibre diameter variability and that it could possibly be a gene marker for improving wool traits.

## Simple Summary

Wool is a valuable natural material, but its quality can vary. This research explored how a gene that encodes a specific wool protein affects some wool characteristics in Chinese Tan sheep. This is a breed known for producing fibre with a unique crimp pattern. Five different variants of the gene *KRTAP13-3* were identified, and this genetic variation was found to be associated with two traits that describe variability in the heterotypic hair fibres of the sheep. This gene may have potential value as a gene marker for assisting with the genetic improvement of wool traits.

## 1. Introduction

Mammalian genomes contain large gene families, including olfactory receptor genes, immunoglobulin genes, zinc finger protein genes, solute carrier protein genes, T-cell receptor genes, ATPase genes, keratin genes, and keratin-associated protein (KAP) genes. The latter two families play a key role in defining the structure and function of the sheep pelage called wool. Wool has unique properties, yet its use is restricted because of variation in its physical and chemical characteristics. This can affect its value. The heritability of many valued wool traits is moderate to high [1,2], and this presents opportunities for genetic improvement. However, achieving this improvement would require a comprehensive understanding of the genetic factors that regulate these traits.

Wool primarily consists of proteins, with the wool keratins forming structures called intermediate filaments. The KAPs create a matrix that embeds these filaments through various interactions [3]. They are classified into the high sulphur (HS) group, the ultra-high sulphur (UHS) group, and the high glycine–tyrosine (HGT) group [3]. While the interactions between the HGT-KAPs and the intermediate filaments are not fully understood, the HS- and UHS-KAPs cross-link with the intermediate filaments via disulfide bond formation [3].

The full set of KAP genes (*KRTAPs*) has yet to be identified in sheep [4]; however, in humans, 89 functional *KRTAPs* across 25 KAP families (KAP1 to KAP13, KAP15 to KAP17, and KAP19 to KAP27) have been identified [5,6,7]. Genes from two of the HS-KAP families, KAP11 and KAP13, are the first to be expressed in hair follicles from humans, with their expression occurring in tandem with the expression of keratins and prior to expression of the first HGT-KAP [5,7,8,9].

The human KAP13 family comprises four members [9]. Recently, three members of the KAP13 family were described in sheep, including how they affected selected wool traits [10]. Despite their chromosomal proximity, these three KAP13 genes exhibited different levels of sequence variation, and they had different associations with variation in wool traits. Specifically, *KRTAP13-1* was less polymorphic compared with *KRTAP13-2* and *KRTAP13-4*. In Chinese Tan sheep, an indigenous breed that produces ‘spring-like’ crimped wool in early life [11], *KRTAP13-1* appeared to be monomorphic, whereas variation in *KRTAP13-2* was associated with fibre diameter standard deviation (FDSD) and the coefficient of variation of fibre diameter (CVFD) for the heterotypic hair fibres (fibres that can change seasonally at times, tending towards being more hair-like and potentially medullated in the summer, but reverting to being wool-like in the winter for warmth) [10]. In contrast, no association was found between *KRTAP13-4* variation and the four fibre diameter-related traits investigated.

Although *KRTAP13-3* has been identified and is polymorphic [12], its effect on wool traits remains unexplored. This study aimed to investigate the variation of *KRTAP13-3* in Chinese Tan sheep, assess its association with four fibre diameter-related traits, and compare these findings with other *KRTAP13-n* family members.

## 2. Materials and Methods

### 2.1. Chinese Tan Sheep Studied and the Measurement of Key Wool Traits

Two hundred and thirty-eight lambs of the Tan breed were studied. These were the offspring of ten unrelated sires, with all the lambs born in the same year, at the same farm. They were all exposed to similar environmental conditions and feeding regimes. All but six lambs (three sets of twins) were singletons at birth, and to avoid possibly confounding subsequent analyses, these six lambs were removed from the study. This left 232 lambs for genetic analysis.

Samples of fibre were collected from each lamb. These were collected from the mid-side region of the lambs near the middle of the Er-mao period (at day 35 postpartum). Next, the fine wool fibres were manually separated from the heterotypic hair fibres in the sample, capitalising on their differences in fibre diameter and length. This was undertaken by spreading each fibre sample on a flannel-coated panel. A stiff plastic card was then used to press the bottom of all the fibres onto the panel, which allowed the longer heterotypic hair fibres to be pulled out of the sample. The process was repeated several times to ensure that the heterotypic hair fibres and the fine wool fibres were fully separated.

The mean fibre diameter (MFD), FDSD, and mean fibre curvature (MFC) of the heterotypic hair fibres and finer wool fibres were then measured. The fine wool samples were evaluated by Pastoral Measurements Limited (Timaru, New Zealand), while the hair samples were measured by the New Zealand Wool Testing Authority (NZWTA, Ahuriri, Napier, New Zealand) using the International Wool Textile Organisation (IWTO) standardised Laserscan method IWTO 12 [13]. The CVFD was calculated from MFD and FDSD by dividing the FDSD by the MFD and multiplying by 100 to express it as a percentage.

Venous blood samples from each lamb were collected onto blotting paper (TFN paper; Munktell Filter AB, Falun, Sweden). These samples were allowed to dry in the air and were then kept in the dark at room temperature until further use. With this blood collection approach, the genomic DNA in the leukocytes becomes bound to the paper, and the DNA was then purified for use in PCR amplification as follows.

A 1.2 mm punch was taken from the blood spot on the TFN blotting paper and placed in a 0.7 mL PCR tube. Each tube contained a 200 µL volume of 20 mM NaOH. These tubes were then incubated at 60 °C for 30 min. Next, the NaOH solution was carefully removed by aspiration, and the disks with attached DNA were equilibrated in 200 µL of TE^−1^ buffer (10 mM Tris-HCl, 0.1 mM EDTA, pH 8.0). This buffer was then removed by aspiration, and the disks were air-dried until used to prime Polymerase Chain Reaction (PCR) amplifications.

### 2.2. PCR Amplification and SSCP Analysis of KRTAP13-3

Two PCR primers were created to amplify a 310 bp region of ovine *KRTAP13-3* that was known to be polymorphic based on the sequences of five ovine *KRTAP13-3* variants in GenBank (Accession numbers JN377429-JN377433). These primers were 5′-GCTCTTCTCTCTACAGTCGA-3′ (forward primer) and 5′-CAGGGAAAATTCATTGGGTGC-3′ (reverse primer), and they were synthesized by Integrated DNA Technologies (Coralville, IA, USA).

The PCR amplification reactions were undertaken in a 15-μL reaction that contained 0.5 U Taq DNA polymerase (Qiagen, Hilden, Germany), 2.5 mM Mg^2+^, 0.25 μM each primer, 150 μM each dNTP, 1× reaction buffer supplied with the enzyme, and the purified genomic DNA on a 1.2-mm TFN card disk, made up to volume with double-deionized water. The amplifications were performed in thermal cyclers (Model S1000; Bio-Rad, Hercules, CA, USA) with an initiating denaturation of 2 min at 94 °C, followed by 35 cycles of 94 °C for 30 s, 60 °C for 30 s, 72 °C for 30 s, and a final extension step at 72 °C for 5 min.

Single-stranded conformation polymorphism (SSCP) analyses were used to examine the PCR amplicons. With this analytical method, which can resolve DNA sequence variation to the single nucleotide level, a 0.7-μL aliquot of each PCR amplification reaction was mixed with 7 μL of gel loading dye (0.025% bromophenol blue, 0.025% xylene cyanol, 10 mM EDTA, and 98% formamide). These solutions were denatured (90 °C for 5 min), and the samples were placed on wet ice prior to being loaded immediately onto 16 × 18 cm, 12% acrylamide:bisacrylamide (37.5:1; Bio-Rad) gels. Protean II xi cells (Bio-Rad) were used for electrophoresis with coolant circulating at 15 °C. The samples were electrophoresed at 320 V in 0.5× TBE buffer for 18 h. Ovine blood samples that produced the DNA sequence variants identified previously by Gong et al. [12] were obtained, and PCR products from these samples were run on each gel as a reference to determine the variants present in each Tan sheep sample.

The polyacrylamide gels were silver-stained in a solution of 10% ethanol, 0.5% acetic acid, and 0.2% silver nitrate for 10 min and then rinsed in distilled water and developed with a solution of 3% NaOH and 0.1% HCOH until dark-staining bands appeared against the yellow background of the gel.

### 2.3. Statistical Analyses

The genetic diversity of *KRTAP13-3* variation was assessed using two genetic parameters: genetic heterogeneity and polymorphism information content (PIC). These parameters were determined using an online calculator (https://www.genecalculators.net/pq-chwe-polypicker.html, accessed on 5 February 2025).

General Linear Models (GLMs) were developed using Minitab version 16 (Minitab Inc., State College, PA, USA) and used to assess the influence of the presence or absence of the *KRTAP13-3* variants detected on the fibre traits, but only for variants with frequencies greater than 5% in the Tan lambs. This was because variants present in only a small number of lambs can bias the analyses if these lambs have extreme phenotypes. Gender and sire were included as fixed and random factors, respectively, in the models.

The model used to examine the effect of variant presence/absence was Y_ijkl_ = μ + V_i_ + Gender_j_ + Sire_k_ + e_ijkl_, where Y_ijkl_ is a phenotype of the ijkl-th sheep (for either MFD, CVFD, FDSD, or MFC), μ = is the arithmetic mean for the characteristic, V_i_ is the effect of the i-th variant (presence and absence), Gender_j_ is the fixed effect of j-th gender, Sire_k_ is the random effect of k-th sire, and e_ijkl_ is the random residual effect. When *p* < 0.05, the associations were considered significant.

## 3. Results

**Figure 1:**
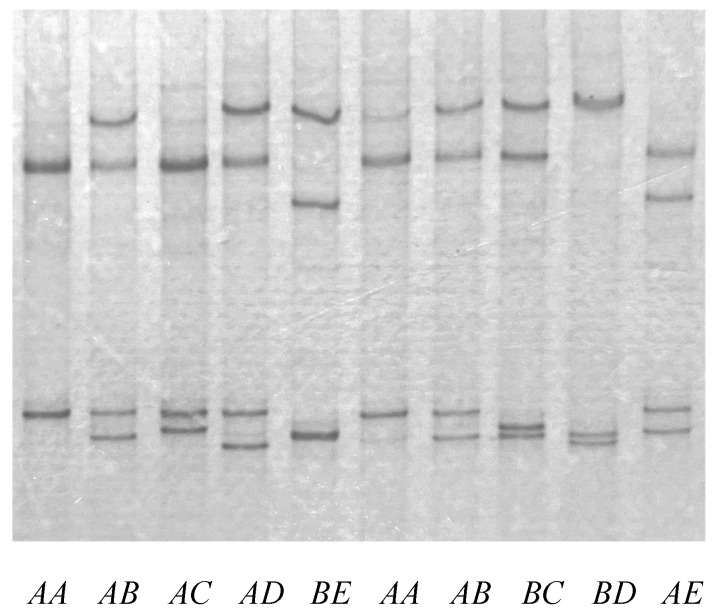
PCR-SSCP patterns of ovine *KRTAP13-3* variants. Five different banding patterns (*A* to *E*), corresponding to five different DNA haplotypes were observed. These occurred in either homozygous or heterozygous forms, with different nucleotide sequences expected to produce different banding patterns on the gels. Each DNA sequence produces two bands that correspond to the two strands of the DNA for any given variant, with heterozygous sheep usually having four bands.

All five variants identified previously [12] were detected in the Chinese Tan sheep using PCR-SSCP (Figure 1). Twelve different genotypes were revealed: *AA* (*n* = 92), *AB* (*n* = 51), *AC* (*n* = 40), *AD* (*n* = 18), *AE* (*n* = 4), *BB* (*n* = 5), *BC* (*n* = 11), *BD* (*n* = 5), *BE* (*n* = 1), *CC* (*n* = 2), *CD* (*n* = 2), and *DD* (*n* = 1). The frequencies of variants *A* through *E* were 64.0%, 16.8%, 12.3%, 5.8%, and 1.1%, respectively. The heterogeneity and PIC of this gene were 54.4% and 50.4%, respectively.

As variant *E* occurred at a frequency under 5%, five sheep carrying this variant were excluded from the association analyses, resulting in a final dataset for modelling analysis of 227 sheep. Among these sheep, variation in *KRTAP13-3* was associated with FDSD and CVFD in heterotypic hair fibres (Table 1). The presence of variant *A* was linked to increased FDSD and CVFD in heterotypic hair fibres, while trends of association were also observed for variants *B* and *D*, which were associated with decreased CVFD. No associations were found with variation in fine wool fibres.

## 4. Discussion

This study identified multiple variants of *KRTAP13-3* in Chinese Tan sheep, but with differences in their frequency distribution compared with the New Zealand (NZ) Romney sheep described by Gong et al. [12]. In the NZ Romney sheep, the common variants (*A*, *B*, and *C*) had similar frequencies, while the rare variants (*D* and *E*) were much less common. In contrast, within the Chinese Tan sheep population, variant *A* was the most common, with variants *B* and *C* occurring at lower frequencies. Variant *D* was less common again, while variant *E* was the rarest.

Within the Chinese Tan sheep population, *KRTAP13-3* exhibited a greater genetic heterogeneity and PIC when compared with *KRTAP13-1* and *KRTAP13-4* [10], but it was comparable to *KRTAP13-2*. This might suggest that the increased diversity of *KRTAP13-3* and *KRTAP13-2* has greater functional effect, with this supported by the observed associations revealed in this study and by Bai et al. [10], respectively. Equally, Bai et al. [10] suggested that *KRTAP13-1* was monomorphic; hence, no effects on wool traits could be investigated. Care is needed in drawing these conclusions though, as both studies are limited in size and involved only a single breed or group of wool sheep. This work needs to be expanded to the more common wool breeds, such as the Merino, and include other important wool traits too.

The associations detected for *KRTAP13-3* in this study are like those found with *KRTAP13-2*, which was also associated with the FDSD and CVFD of heterotypic hair fibres [10]. *KRTAP13-3* is located approximately 11.2 kb from *KRTAP13-2*, suggesting that the proximity of the two genes underpins a common effect. However, *KRTAP15-1*, a member of a different gene family is also co-located, being approximately 15.0 kb from *KRTAP13-3*. In Merino × Southdown-cross sheep, variation in *KRTAP15-1* was strongly associated with wool yield, although an association with FDSD was also observed [14]. Since these studies involved different sheep breeds and measured different wool traits, comparisons should again be made with caution, and it remains a challenge to determine whether the associations detected are due to the genes themselves, linkage with nearby genes, or the effect of being part of a gene family that may have coordinated activity; albeit, the differences between the four *KRTAP13* genes would suggest that the latter is unlikely. Further investigation into the associations of *KRTAP15-1* and *KRTAP13-n* and within the same populations may provide additional insight.

The association between *KRTAP13-3* and FDSD and CVFD, but not with MFD, suggests that variation in the gene principally affects the uniformity of the measured fibre diameters, rather than the average diameter of the fibres sampled. The exact mechanism underlying these associations remains unclear, but one possible explanation is that *KRTAP13-3* may influence wool fibre ellipticity. Wool fibres typically have an elliptical rather than a circular cross-section, and studies have shown that higher FDSD and CVFD are linked to increased ellipticity [15]. Alternatively, the variation in FDSD and CVFD may reflect along fibre variation in diameter, with this possibly reflecting changes in follicle activity over time; albeit, this too needs further study.

Interestingly, the associations are only observed for the heterotypic hair fibres and not the fine wool fibres. One possible explanation is the differential expression of *KRTAP13-3* between these two fibre types. Heterotypic hair fibres, which are coarser and exhibit greater variability in diameter, may express *KRTAP13-3* differently compared with fine wool fibres, potentially leading to a more pronounced impact on fibre diameter uniformity. The structural differences between coarse and fine fibres could affect how KAP13-3 protein influences fibre ellipticity and/or uniformity. Coarser fibres, with their large surface area, are generally more susceptible to external forces, which might further amplify the gene’s effect on fibre structure. In contrast, fine wool fibres, with their more consistent diameter and reduced susceptibility to external forces, may show a diminished impact of the gene on their structure and, hence, not reveal the same associations. This suggests that more research is needed to validate these findings and to further explore the differential expression of *KRTAP13-3* in various fibre types. Investigating how *KRTAP13-3* expression varies among different fibre types and under different conditions will provide a better understanding of its role in fibre traits and confirm the observed associations.

The key sequence difference between *KRTAP13-3* variants *A* and *B* or *D* is the c.242G/A substitution, which results in the amino acid change p.Arg81Gln [12]. The substitution of a positively charged arginine with a neutral glutamine could alter the protein’s structure and its interactions with other proteins, potentially leading to effects on increased FDSD and CVFD in heterotypic hair. This too needs further investigation at the functional level.

## 5. Conclusions

Five previously identified variants of *KRTAP13-3* were detected in the Chinese Tan sheep studied using PCR-SSCP. The most common variant (*A*) was linked to increased FDSD and CVFD. No associations were found with variation in the fine wool fibres from the Chinese Tan sheep. These findings suggest that *KRTAP13-3* plays a role in regulating heterotypic hair fibre diameter variability.

## Figures and Tables

**Table 1:** Association of *KRTAP13-3* variants with wool traits in Chinese Tan sheep.

Fibre Type	Fibre Trait ^1^	Variant	Present		Absent		*p*-Value ^2^
			Mean ± SE	*n*	Mean ± SE	*n*	
Fine wool	MFD (µm)	*A*	16.7 ± 0.17	201	16.7 ± 0.33	26	0.429
		*B*	16.9 ± 0.23	72	16.6 ± 0.18	155	0.133
		*C*	16.8 ± 0.25	55	16.6 ± 0.17	172	0.404
		*D*	16.4 ± 0.31	26	16.7 ± 0.18	201	0.349
	FDSD (µm)	*A*	4.2 ± 0.12	201	4.0 ± 0.24	26	0.429
		*B*	4.3 ± 0.17	72	4.2 ± 0.13	155	0.558
		*C*	4.2 ± 0.18	55	4.2 ± 0.13	172	0.799
		*D*	4.0 ± 0.23	26	4.2 ± 0.13	201	0.371
	CVFD (%)	*A*	25.0 ± 0.58	201	23.9 ± 1.12	26	0.285
		*B*	25.1 ± 0.78	72	24.9 ± 0.61	155	0.809
		*C*	24.8 ± 0.86	55	25.0 ± 0.59	172	0.852
		*D*	24.3 ± 1.05	26	25.1 ± 0.62	201	0.473
	MFC (^o^/mm)	*A*	63.9 ± 1.14	201	64.0 ± 2.21	26	0.961
		*B*	62.7 ± 1.54	72	64.4 ± 1.20	155	0.245
		*C*	65.2 ± 1.68	55	63.6 ± 1.16	172	0.303
		*D*	65.9 ± 2.07	26	63.3 ± 1.22	201	0.237
Heterotypic hair	MFD (µm)	*A*	29.7 ± 0.34	201	30.0 ± 0.67	26	0.564
		*B*	29.7 ± 0.46	72	29.7 ± 0.35	155	0.999
		*C*	29.7 ± 0.51	55	29.7 ± 0.34	172	0.877
		*D*	30.1 ± 0.62	26	29.6 ± 0.37	201	0.432
	FDSD (µm)	*A*	8.3 ± 0.15	201	7.7 ± 0.31	26	**0.033**
		*B*	8.1 ± 0.21	72	8.4 ± 0.16	155	0.105
		*C*	8.3 ± 0.23	55	8.3 ± 0.16	172	0.906
		*D*	8.0 ± 0.28	26	8.4 ± 0.17	201	0.261
	CVFD (%)	*A*	28.1 ± 0.45	201	25.7 ± 0.90	26	**0.005**
		*B*	27.2 ± 0.63	72	28.2 ± 0.48	155	*0.094*
		*C*	27.8 ± 0.69	55	28.0 ± 0.47	172	0.789
		*D*	26.7 ± 0.84	26	28.3 ± 0.50	201	*0.082*
	MFC (^o^/mm)	*A*	46.6 ± 0.72	201	45.3 ± 1.43	26	0.309
		*B*	46.2 ± 1.00	72	46.6 ± 0.76	155	0.609
		*C*	46.8 ± 1.09	55	46.5 ± 0.74	172	0.742
		*D*	45.8 ± 1.33	26	46.7 ± 0.79	201	0.524

^1^ MFD—mean fibre diameter; FDSD—fibre diameter standard deviation; CVFD—coefficient of variation of fibre diameter; MFC—mean fibre curvature. ^2^ Values of *p* < 0.05 are shown in bold; values of *p* < 0.10 are shown in italics.

## Data Availability

The raw data supporting the conclusions of this article will be made available by the authors on request.
